# An improved method for assessing mismatches between supply and demand in urban regulating ecosystem services: A case study in Tabriz, Iran

**DOI:** 10.1371/journal.pone.0220750

**Published:** 2019-08-15

**Authors:** Vahid Amini Parsa, Esmail Salehi, Ahmad Reza Yavari, Peter M. van Bodegom

**Affiliations:** 1 School of Environment, College of Engineering, University of Tehran, Tehran, Iran; 2 Institute of Environmental Sciences (CML), Leiden University, Leiden, Netherlands; International Institute for Applied Systems Analysis (IIASA), AUSTRIA

## Abstract

Regulating ecosystem services provided by urban forests are of great importance for the quality of life among city dwellers. To reach a maximum contribution to well-being in cities, the urban regulating ecosystem services (URES) must match with the demands in terms of space and time. If we understand the matches or mismatches between the current urban dwellers’ desired quality conditions (demand) and the supply of URES by urban forests (UF) in the cities, this will facilitate integrating the concepts of ecosystem services in urban planning and management, but such an assessment has suffered from major knowledge limitations. Since it is complex and problematic to identify the direct demands for URES and the spatiotemporal patterns therein, improving the demand indicators can help to determine the actual requirements. In this paper, a methodological approach based on indicators is presented and demonstrated for two important URES: air quality improvement and global climate change mitigation provided by urban trees and shrubs. Four air quality standards and greenhouse gas reduction targets were used and compared to supplies of the URES in Tabriz, Iran. Our results show that the mean contribution of the URES supply to air quality standards and greenhouse gas reduction targets is modest. Hence, in Tabriz, there is a strong mismatch between demand and supply. Mismatches at the city scale will have to be reduced by both a reduction in pollutant emissions and an increased provisioning of URES supply through urban greenery. The presented assessment approach and the results for Tabriz make it explicit how different the demands and supplies of the two studied URES are, and we expect similar mismatches in many other cities. Therefore, our approach, relatively simple but still realistic and easy-to-apply, can raise awareness about, and the utility of, the ecosystem services concepts for urban planning and policymaking.

## Introduction

Ecosystem services (ES) encompass a diverse concept [1] which has gained growing attention among scientists so as to inform decision makers to support more sustainable use of ecosystems [2–4]. Its attractiveness lies in its interdisciplinary nature which comprises both socio-economic and natural sciences. Consequently, ES have been called the missing link or the bridge between human well-being (social systems) and ecosystems (ecological system) [5–7]. Part of the attractiveness also lies in the ambiguity of the concept [8]. These properties of complexity and ambiguity have, however, hampered the development of ES assessments and prevented embedding ES deeply in environmental decision-making processes [4,9–11]. Therefore, for more practical applications, the tools need to be made more applicable for environmental resources management [4,12,13]. This is particularly urgent for urban ecosystems [14,15]. Cities are more challenging than natural bodies because they consist of complex, intense, and spatially diverse interactions between socio-ecological systems. Urbanization creates both solutions and problems for urban sustainability [16–20].

The role of urban ecosystems, and especially UF, in providing benefits and services to urban residences has been stressed frequently, and it is necessary to incorporate ES in urban planning and policies [14,21,22]. Some ES generated by urban ecosystems are delivered locally to be enjoyed directly by urban residences (e.g. air purification), and some are even directly tangible to residents (e.g. climate change regulation through carbon sequestration) [15,23,24].

Even so, the complexity of ES concept and urban areas has prevented making ES operational in urban planning and policy-making processes so far [15]. One of the main obstacles in applying ES in decision-making is conceptualizing the delivery of ES to the society [25,26]. This requires the development and use of appropriate indicators [8]. While the development of ecosystem service indicators is an active field of research, most indicators refer to the (potential) supply of ecosystem services, and few studies make a distinction between the capacity of the ecosystem to produce a service and the societal demand for it (ES supply) [9,27]. In order to develop a sustainable management of ES, however, it is essential to assess both supply and demand so as to be able to analyse the matches or mismatches between the supply and demand of ES [2,28,29]. Despite the importance of considering ES demands in assessments [1,30], few ES assessments have integrated the indicators of the mismatches between the ES supply and demand.

This lack of evaluation applies especially to urban areas [9,14,31], for which quantifying the ES demand (especially for the URES) is complicated due to the complexity in the direct relations between ES and human benefits and the fact that indicators tend to relate to ES supply instead of ES demand [9]. It is challenging and difficult to measure the demand for URES [32], and up-to-date, comprehensive indicators for URES demand are barely studied. As a consequence, URES are depreciated and disintegrated in planning [25], except in a few cases (see [13,33]). Regulating services are of particular importance to cities [23,34], especially two of those services–air purification and global climate regulation (through carbon sequestration) [16]–because the significant environmental problems in most cities, especially in the developing countries, are air pollution and climate change, which cause adverse effects on human health and well-being [35–40].

Urban areas can be considered both as a hotspot for greenhouse gas (GHG) emissions (cities are responsible for more than 70% of the global CO_2_ emissions [41–45]) and a carbon sink (sequestrating carbon through urban forest and soil) [46,47]. Thus, while urban areas occupy a small proportion of the landmass, they have an essential role in the Global Carbon Cycle [43,46]. Moreover, cities are more vulnerable to climate change [48,49]. Therefore, urban areas should be taken into account in global climate change mitigation and adaptation efforts [47,50–53]. One way to mitigate the climate change in cities is through their regulating ES especially those provided by urban trees and shrubs (i.e. climate change regulation service through carbon sequestration and storage by UF) [49,52,54–56]. Sequestrating carbon by UF and consequently storing it in UF biomass contributes to balancing the global carbon budget and then plays a significant role in determining the concentration of CO_2_ in the urban atmosphere [57]. Therefore, UF can be considered as an effective tool in mitigating climate change [45,58–60].

Urban air quality deterioration caused by high concentration of air pollutants (e.g. NO_x_, SO_2_, O_3_, CO and PM_10 and 2.5_) is considered as a key environmental problem which induces adverse effects on human health (it is responsible for about 11.6% of all global deaths) [35–38,61]. Therefore, there is a need for opportunities to mitigate and reduce urban air pollution [62,63]. Planting and maintaining UF is one of the most considerable strategies developed and evaluated to mitigate, adapt, and overcome urban air pollution problems [20,38,64–67].

There is a growing body of literature that indicates the important contribution of urban forests in improving the environmental quality (e.g., clean air) within cities [68–70]. However, the URES provision by urban forests is rarely compared to the demands or the desired quality conditions in cities [14]. The assessment of the matches or mismatches between the URES supply and demand helps to identify the biophysical capacity of a specific area to produce the URES, to evaluate the sustainability of URES delivery and to analyze to what extent the demand (needs of people) can be met by the current urban forests’ characteristics and functions (without any decrease or damage to the ES capacity) [3,25]. Thus, it is essential to further develop approaches for such assessments for cities so as to facilitate their utility in urban environmental decision-making processes [25].

Therefore, in this study, we aimed to assess the matches and mismatches between the supply and demand of two urban regulating ES in which UF plays a critical role: air purification and global climate regulation. Based on this analysis, we explore the possible contribution of URES supply provided by UF to meeting policy targets and environmental quality standards (EQS) in an urban context. As indicators for ES demand, we use the EQS-based approach [9,14,71]. We tested our approach in a case study in Tabriz, Iran. The obtained results can help to identify the actual contribution of UF to human wellbeing and to show the utility of the concept of ecosystem services for urban planning and policymaking.

## Materials and methods

### Study area

**Fig 1:**
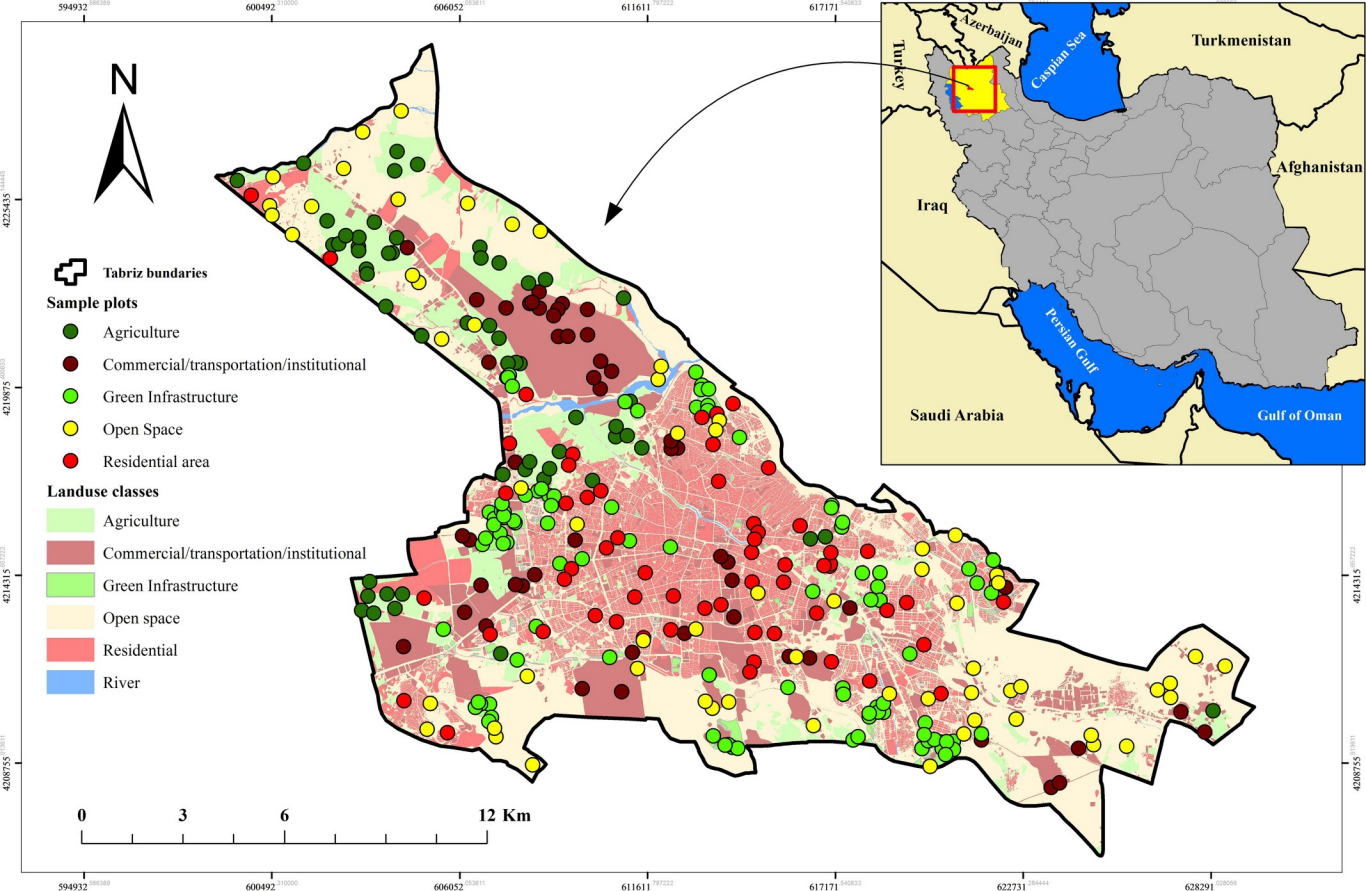
Location of Tabriz municipality, land use classes, and sample points.

**Fig 2:**
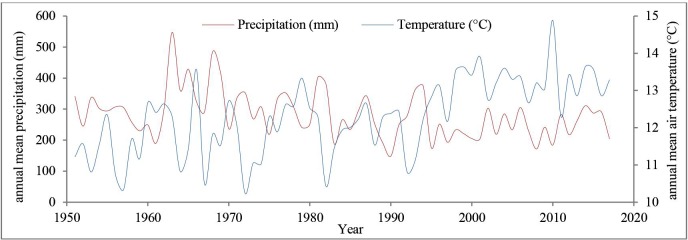
Historical trends in annual mean temperature and precipitation in Tabriz (1951–2017)—(Data source: [73]).

This study was carried out in Tabriz (244.8 km^2^; 1.56 million inhabitants with 49.8 thousand households) [72], northwestern Iran, in East Azerbaijan Province (Fig 1). It occupies the eastern and southeastern lands of the Tabriz plain and is surrounded by the Einali and Sahand mountains. Its average elevation is 1351 m above sea level. The climate is semiarid, characterized by relatively hot summers (up to 42°C) and very cold winters (down to -25°C). The annual mean air temperature is about 12°C, but it has experienced the incremental trend by about 2°C from 1951 (11.21°C) to 2017 (13.28°C) (Fig 2). The annual mean precipitation has decreased from 341.2 mm in 1951 to 204.3 mm in 2017 (the overall annual mean precipitation in this period is about 3111.1 mm) (Fig 2) [73]. Rainfall events tend to be intensive due to the proximity of mountains. Land uses range from agricultural to urban. Urban parks and green spaces are mainly outside the city center and share similarities in their recreational use and the common presence of grass cover and occurrence of deciduous and evergreen trees [67].

The economic growth and centralization have caused the influx of industries and people into the metropolitan area of Tabriz and have extended the city size by about 35 times in a century. This has also caused land use changes; about 4715 ha of green spaces were converted to other land uses during the last three decades [74] and caused environmental quality deterioration [75,76].

The relatively high anthropogenic pollutant emissions (since it is considered the “commercial and industrial heart” of the northwestern Iran), the unique topographic position–being surrounded by mountains that act as a trap for pollutants [75,76] occasionally aggravates air pollution by contributing to thermal inversions–and the meteorological conditions of Tabriz make it one of the worst among the major Iranian cities in terms of urban air quality problems.

**Fig 3:**
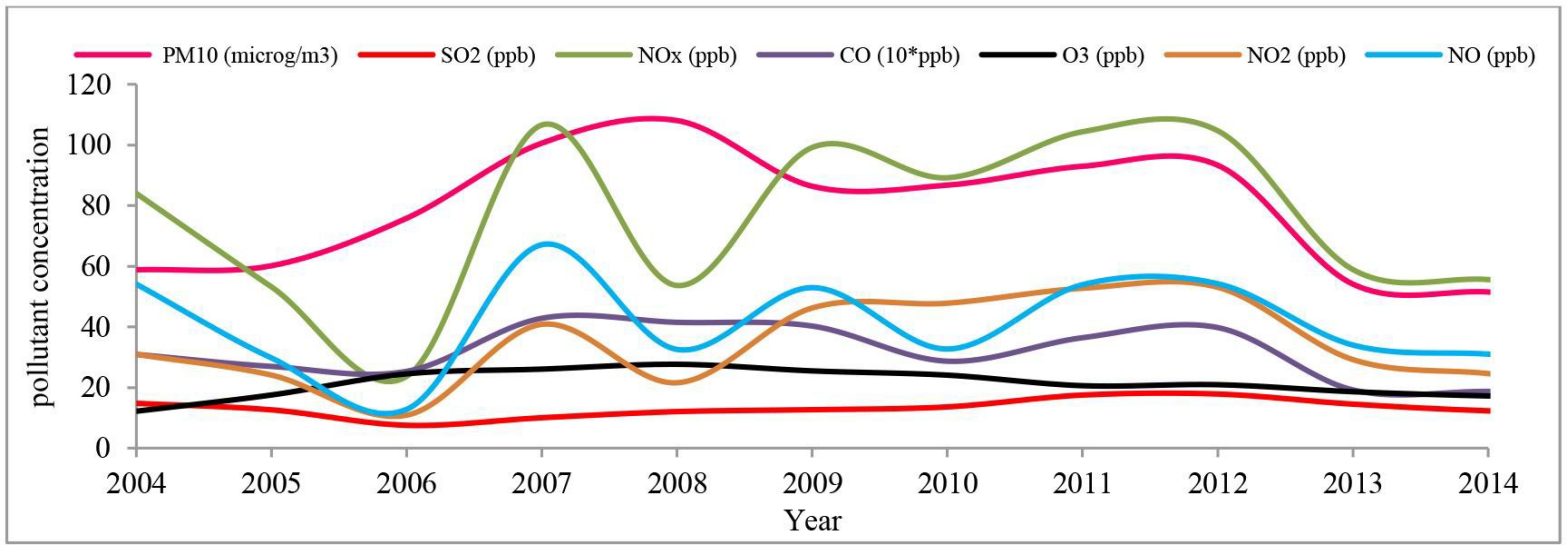
Dynamic trends in the annual mean concentration of air pollutants in Tabriz (2004–2104)—(Data source: [80]).

Air pollution in Tabriz is considered as the most critical environmental problem [77], such that as the statistics indicate, only 19% of the year had seen clean air in 2014 [78]. CO and PM are among the main air pollutants in Tabriz [79]. The dynamic trends in the annual mean concentration of air pollutants show that the air quality has partially improved from 2012 (Fig 3).

## Methodology

To enhance our ability to quantify and, ultimately, make ES information more accessible to decision-makers, we have to make a clear distinction between, and have a clear definition of, the key elements in the ES delivery process; capacity, flow and demand, so as to assess the mismatch or match between the ES supply and demand [10,13,81]. In this paper, we conceptualized *supply* as the “ES flows or biophysical impact of the ES on the environment in or surrounding the area [82]” and *demand* as “the required or desired amount of ES delivered by the society” [25,33,83].

URES supply can be measured directly by determining the amount of ES delivered by urban dwellers in a particular area (in this case, Tabriz) within a given time period (one year in this case) [13,27]. In this study, we used the i-Tree Eco model to assess the supply of two important services provided by UF, i.e. “air quality improvement” and “global climate change mitigation through carbon storage and sequestration”.

Since there is no clear end product of URES and it is challenging to measure the demand for it [32], the environmental quality standards (EQS) have been used as an amenable indicator and measure for service demand [14,84,85]. The main advantage of applying environmental quality as a proxy for URES is that it is meaningful to people, is readily measured [25], relies on scientific evidence [14] and that any change in the environmental quality can be valued in monetary terms [32]. Therefore, we adopted a novel methodological approach using EQS as proxy indicators for the demand side.

We assessed the mismatches between the URES supply and demand following four main steps: 1) assessing the supply of URES, 2) selecting environmental quality standards, 3) assessing the demand for URES and 4) identifying matches or mismatches between the URES supply and demand:

*Step1*: *Assessing the URES Supply*: The supply of URES was defined as the amount of pollutants (i.e. PM_2.5_, O_3_, NO_2_, SO_2_ and CO) removed annually and the annual carbon sequestration per hectare by UF, which are respectively for air quality improvement and climate change regulation supply.

**Fig 4:**
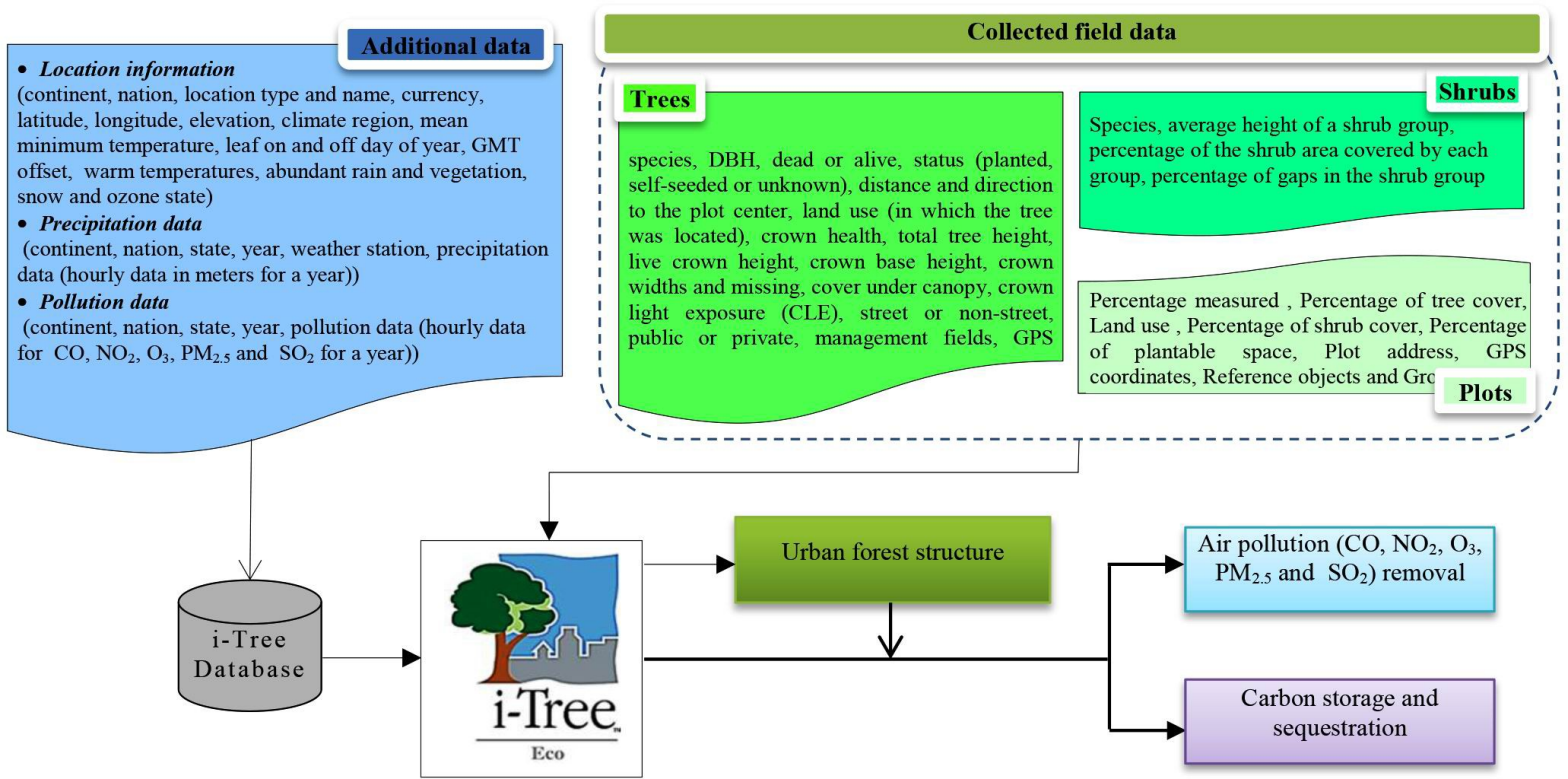
The methodological steps to quantify URES supply through i-Tree Eco model.

As one of the most commonly applied models [35], the i-Tree Eco model was used to quantify and estimate the supply of URES by UF. As the basic i-Tree process is Structure-Services [86], it first estimates the structural and compositional characteristics of UF (urban trees and shrubs) based on collected field data. Then it integrates additional data (i.e., location information, precipitation and pollution data) and the estimated UF structural data, to quantify air pollution removal and carbon sequestration and storage by UF [87] (see Fig 4). Here are the three main steps followed to quantify the supply of the studied URES by i-Tree Eco:

*Adapting i-Tree Eco to the studied area*. i-Tree Eco was specifically developed for the US, so it has to be adapted for the users outside the US and requires the integration and submission of additional data to the i-Tree Database [88]. In this regard, the additional data are collected and introduced into the i-Tree Database web application to conduct and complete our project. Pollution data for O_3_, SO_2_, NO_2_, CO and PM_2.5_ were obtained in an hourly format for a complete calendar year (2015) from the Department of Environment, Iran [80] from an atmospheric monitoring station (located in 38.07° N and 46.29° E). Hourly precipitation data (meters per hour) of the synoptic station of Tabriz (located in 38.13° N and 46.23° E) were obtained for the same year (2015) from Iran Meteorological organization [73]. These data were submitted to the i-Tree Database.

Also, other required weather data were obtained automatically by i-Tree Eco form the selected station (synoptic station of Tabriz). The US Forest Service vetted all this data and incorporated them into the new version of i-Tree Eco.

*Assessing the structure and composition of UF*: Following the i-Tree Eco protocols [87–89], the required field data (i.e. trees, shrubs and plots data) were collected from 330 plots (randomly distributed among the land use classes using Random Points Generator of Arc GIS 10.4.1) through a field survey conducted from 5^th^ of June to 2^nd^ of October, 2017. This number of plots would be sufficient to achieve a standard error of about 10% for the entire city [90].

Using the pre-stratification method, the study area was divided into smaller units based on land use classes. The original land use map was obtained from the municipality of Tabriz for the year 2017 (with 1:25000 scale) and then reclassified into seven classes (Fig 1).

The i-Tree Eco model processes the imported field data to assess the urban forest structure (i.e. summary of structure by species and strata–e.g. the number of trees, LA, leaf biomass, tree dry weight biomass–, summary of population–e.g. the percentage of population–, distribution of species by DBH classes, diversity indices and ranges of species) [86,87].

*Estimating the provision of URES (supply side)—Air quality improvement*: i-Tree Eco estimates the amount of pollution removed by UF within a year through dry deposition modeling for O_3_, SO_2_, NO_2_, CO and PM_2.5_ and also calculates the hourly air quality improvement based on tree-cover data, hourly weather, and pollution data [86,87].

The hourly air quality improvement per unit of tree cover through the dry deposition of the pollutants I_unit_ (%) was calculated as [91]:

$$I_{unit}=\frac{F}{F+M_{total}}\times 100$$ (1)

Where *F* is the pollutant flux (*gm*^−2^*h*^−1^) and *M*_*total*_ is the total air pollutant mass per unit tree cover (*gm*^−2^*h*^−1^). The hourly air quality improvement for the total tree cover (*I*_*total*_) equals to [91]:

$$I_{total}=\frac{F\times \frac{T_{c}}{100}}{F\times \frac{T_{c}}{100}+M_{total}}\times 100$$ (2)

Where *T*_*c*_ = total urban tree cover (%). (For a more detailed description, see Hirabayashi et al., 2015).

Air pollutant concentration change was calculated as [91]:

$$\Delta C=\frac{C}{1-\frac{I_{total}}{100}}-C$$ (3)

Where Δ*C* is air pollutant concentration change (*gm*^−2^*h*^−1^) and *C* is the air pollutant concentration (*gm*^−2^*h*^−1^).

The expected annual mean levels of air pollution without air purification by urban trees or shrubs (*μg m*^*−3*^) is calculated as the annual mean concentration of pollutants (*μg m*^*−3*^) without considering UF air pollution removal.

*Carbon storage and sequestration*: The urban tree carbon storage was estimated through multiplying the tree biomass by a factor of 0.5. The biomass for each measured urban tree was quantified using the allometric equations from the literature [49,92]. The above-ground biomass, which was estimated based on DBH, tree height and tree condition [86], were converted to the total tree biomass by applying the common root-to-shoot ratio of 0.26 [93]. Also, as the deciduous trees lost their leaves annually, the total stored carbon was estimated by multiplying total tree dry weight biomass by 0.5 [49,94]. Dry-weight biomass for each tree was obtained by multiplying the computed fresh-weight biomass by species- or genus- specific-conversion factors of 0.48 for conifers and 0.56 for hardwoods (see Nowak et al. 2002b). The biomass of urban open-grown and maintained trees–which may have less above-ground biomass–predicted through forest-derived biomass equations, was adjusted by applying a 0.8 factor [86,92]

As the urban tree DBH increases according to an estimated annual growth rate (i.e. the tree biomass increase determines carbon sequestration), the annual carbon sequestration of each urban tree was estimated by contrasting the carbon storage in the current year (X) with carbon storage in the following year (X+1) [49,94].

*Step 2*: *Selecting EQS*: The Environmental quality standards (EQS) are related to regulating processes or ecosystem states and the demand for them is often based on concentration or reduction indicators [9]. In this study, greenhouse gas (GHG) emission targets and the deviations in air quality standards served as demand indicators, respectively, for global climate regulation and air quality improvement.

Global climate regulation targets have mainly been set at the global scale, yet urban GHG emission reduction targets can be considered the desired condition at the city scale [13,14]. As there is no GHG emission target specifically for the city of Tabriz, we applied the Iranian reduction target (to reduce GHG emissions by 4 and 12% by 2030 respectively through unconditional and conditional mitigation action) as the desired condition [95].

EQS for air quality regulation of PM_2.5_, O_3_, NO_2_, SO_2_ and CO were derived from the following four standards: 1) WHO air quality guidelines [96], 2) EU Air Quality Directive [97], 3) the National Ambient Air Quality Standards of the Environmental Protection Agency (EPA) of the United States [98] and 4) the Iran air quality standard [99]. WHO’s reference values are more stringent than the Iranian ones, but only the Iranian air quality standard is legally binding within the Iranian cities (**Table 1**). By considering all the four standards, an appropriate balance was created.

**Table 1:** Selected environmental quality standards (EQS) for assessing the matches and mismatches between urban regulating ecosystem services (URES) supply and demand.

URES	EQS				
**Global climate regulation**	Iranian national Mitigation of Greenhouse Gases Target: 1- Unconditional; 4% by 2030 (through Business as Usual (BAU) scenario) 2- Conditional Mitigation Action; 12% by 2030 (the potential of mitigating additional GHGs emission up to 8% against the BAU scenario through additional mitigation actions)				
**Air quality regulation**	Pollutants	Reference values of selected standards			
		WHO	EU	EPA	Iran
	PM_2.5_	10 μg/m^3^ (annual mean)	25 μg/m3 (annual mean)	12 μg/m^3^ (annual mean)	10 μg/m^3^ (annual mean)
	O_3_	100 μg/m^3^ 8-hour mean	120 μg/m^3^ (Maximum daily 8 hour mean)	159 ppm (8-hour mean)	12 μg/m^3^ (annual mean)
	NO_2_	40 μg/m^3^ (annual mean)	40 μg/m^3^ (annual mean)	0.053 ppm (107 μg/m3) (annual mean)	100 μg/m^3^ (annual mean)
	SO_2_	50 μg/m^3^ (annual mean)	20 μg/m^3^ (annual mean)	79 μg/m^3^ (annual mean)	80 μg/m^3^ (annual mean)
	CO	-	10 mg/m^3^(Maximum daily 8 hour mean)	9 ppm (8-hour) 35 ppm (1-hour)	35 ppm (1-hour) 9 ppm (8-hour)

*Step 3*: *Assessing URES demand*: The demand for URES was defined as the extent to which the ecological pressures need regulation (e.g. purification by UF) in order to fulfill the defined EQS. For air quality regulation, the ecological pressures were defined as the air pollutant concentration, as available for PM_2.5_, O_3_, NO_2_, SO_2_ and CO from the air pollution monitoring stations of the Iranian Department of Environment. The demand indicators for air quality regulation were estimated by relying on each pollutant concentration in relation to the reference values of the selected standards (e.g., remnant air pollution) [14].

The demand indicator for climate change mitigation was estimated based on measured annual GHG emissions (t CO_2_/ha year) [9]. Since there is no GHG emission data specifically for Tabriz, the total GHG emission for the country (Iran; Gt CO_2_-eq) was adapted from the PBL Netherlands Environmental Assessment Agency report on trends in global CO_2_ and total GHG emissions [100] and downscaled to the city of Tabriz based on the number of inhabitants.

*Step 4*: *Assessing the matches or mismatches between URES supply and demand*: The matches and mismatches were defined as the difference between ES supply and demand [1]. If an unsatisfied or remaining demand exists (the supply side not totally meeting the demand side [14]) within the defined area and period, the mismatches between ES supply and demand are recognized [101]. As long as the demand is met without any decrease in the future capacity of URES provision (i.e. tradeoffs), the URES provision would be sustainable. In contrast, URES provision is not sustainable if the demand cannot be met by the existing URES supply or capacity [14,101], or it when involves losing or degrading other ES in return for fulfilling that demand (tradeoffs) [25]. For example, if the urban air quality consistently fails to meet the ambient air quality standards, the flow of air purification ES from UF will be considered unsustainable, reflecting a mismatch.

Demands should be assessed via the same measurement units as the supply, so that we can identify the matches or mismatches, which are either balance, over- or under-supply [13,14,71]. Based on the EQS-based approach adopted in this study to assess the URES matches or mismatches, URES mismatch is identified when the standards or target values specified by the abovementioned EQS are not met through the supply of URES by UF, or, in other words, when the demand side is not entirely met by the current supply provided by UF. On the contrary, if the supply side fulfills the demand, matches are expected. It is also useful to assess the contribution of the URES supply in EQS’ complaisance (e.g., air quality improvement) [14].

For air pollutants, for which an annual mean value of EQS exists, mismatches occur when the air pollution levels exceed the EQS values despite air quality improvement by UF [14]. Pollutants for which the EQS were based on 1-hour or 8-hour mean values were compared to the corresponding hourly or 8-hourly air pollution concentrations. A significant mismatch was indicated if the “air quality improvement compensates only 0–20% of the corresponding exceedance”. Likewise, a modest mismatch was defined as a value compensating more than 20% of the exceedance.

**Fig 5:**
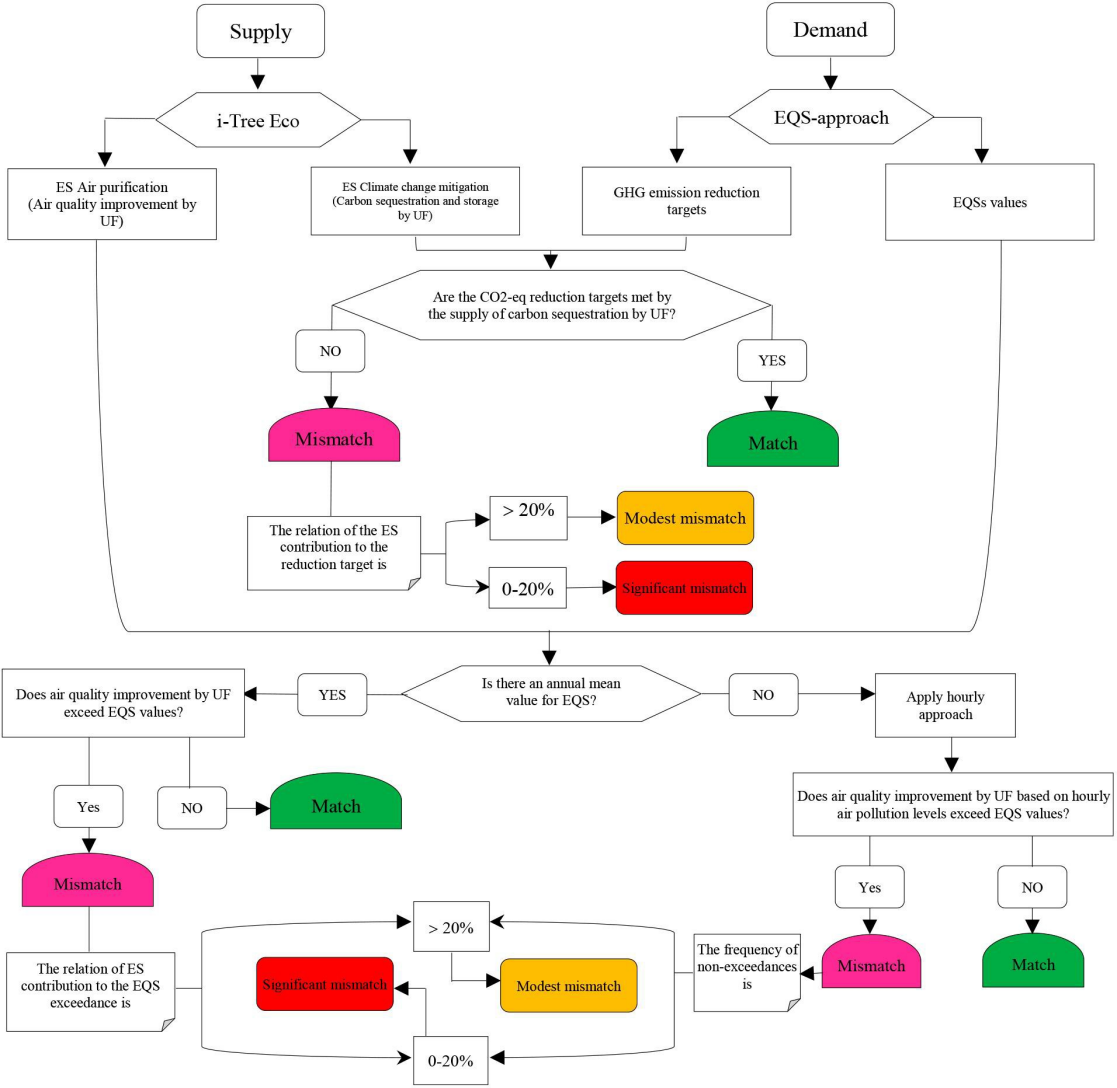
Flowchart for identifying and assessing matches and mismatches between the supply and demand of urban regulating ecosystem services (URES).

Similarly, for global climate change URES, if a contribution of UF to sequestering carbon to comply with the GHG (CO_2_-eq) reduction targets is lower than 20%, it is a significant mismatch and if it is between 20–100%, it is defined as a modest mismatch. Fig 5 presents the entire framework applied in this paper.

## Results

### Urban forest structure

The urban forest of Tabriz had a total of 48 species with an estimated 1,928,000 trees (standard error of about 12.3%) with a tree cover of 9.4% which provided 83.73 Km^2^ of leaf area. The three most common species were *Robinia pseudoacacia* (12.5%), *Fraxinus excelsior* (9.8%), and *Elaeagnus angustifolia* (8.0%). The overall tree density in Tabriz was 79 trees per hectare (the highest tree densities occurred in green spaces followed by residential areas and open spaces).

The potential of the top ten species regarding the provision of air quality improvement and carbon storage and sequestration is shown in Table 2. While these ten species collectively constitute 69.2% of the total number of trees, they were responsible for 61.54%, 61.99% and 64.08% of total carbon storage, annual net carbon sequestration and pollution removal, respectively (Table 2).

**Table 2:** Top ten species due to the provision of air quality improvement and carbon storage and sequestration.

Species	Trees		Carbon Storage		Net Carbon Sequestration		Pollution Removal	
	Number	%	ton	%	ton/yr	%	ton/yr	%
*Robinia pseudoacacia*	240590	12.48	9317.89	14.38	1486.86	16.01	26.29	13.14
*Fraxinus excelsior*	188821	9.80	6967.33	10.75	910.39	9.80	17.72	8.86
*Elaeagnus angustifolia*	153675	7.97	3405.93	5.26	701.52	7.55	15.18	7.59
*Cupressus arizonica*	130009	6.74	3002.26	4.63	312.29	3.36	17.44	8.72
*Ulmus carpinifolia 'Hollandica'*	128342	6.66	3875.48	5.98	271.97	2.93	8.46	4.23
*Ailanthus altissima*	107713	5.59	2072.22	3.20	431.49	4.65	6.43	3.21
*Pinus nigra*	94402	4.90	1930.57	2.98	285.83	3.08	11.72	5.86
*Vitis vinifera*	82417	4.28	1348.71	2.08	287.43	3.10	4.17	2.08
*Pinus eldarica*	73773	3.83	1202.43	1.86	183.2	1.97	7.65	3.82
*Robinia pseudoacacia 'Umbraculifera'*	69096	3.58	885.40	1.37	253.2	2.73	3.36	1.68
*Morus alba*	63579	3.30	5861.11	9.05	632.4	6.81	9.78	4.89
**Sum of the top ten species**	**1332417**	**69.12**	**39869.33**	**61.54**	**5756.58**	**61.99**	**128.20**	**64.08**
**Total**	**1927566**	**100**	**64788.12**	**100**	**10652.84**	**100**	**200.06**	**100**

### Supply and demand for air quality improvement

**Fig 6:**
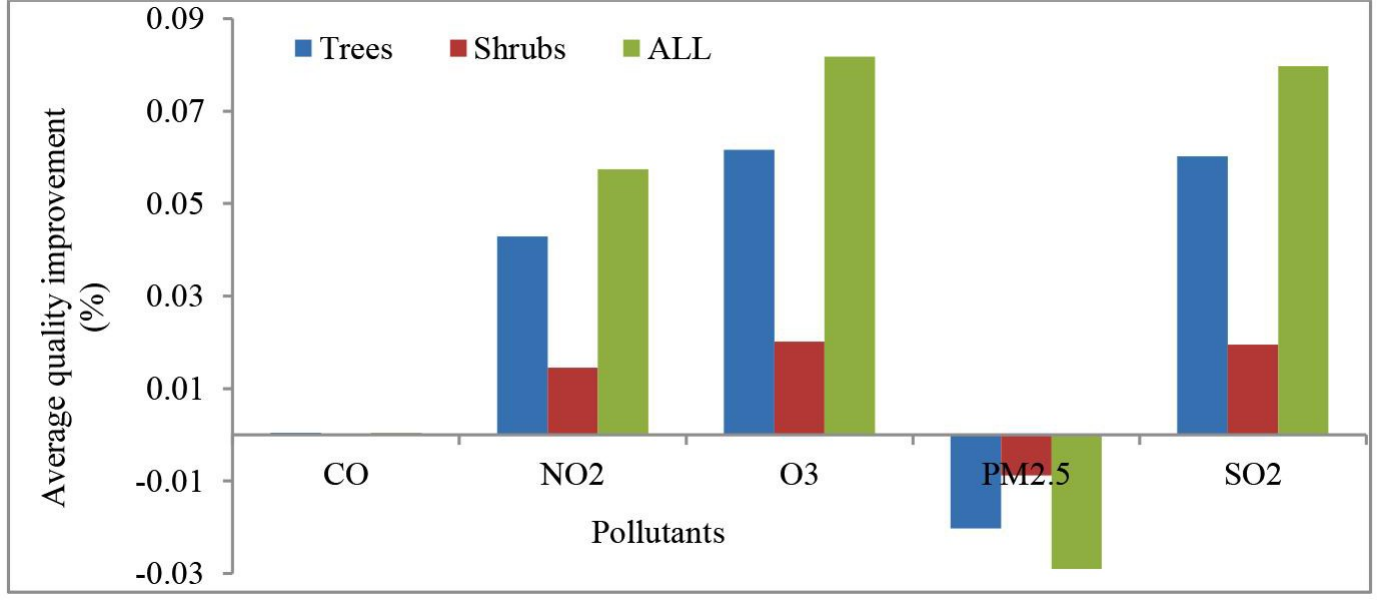
Average percentage of air quality improvement by urban trees and shrubs at the city scale.

The existing urban trees and shrubs in Tabriz were estimated to remove 238.4 t of the air pollutants in 2015. Air quality improvements based on URES supply showed the highest average percentage of air quality improvement for O3 and the lowest value for CO (Fig 6). The results also showed a negative value for PM_2.5_ (Fig 6) which means that the urban forests in Tabriz had a negative contribution in meeting the corresponding EQS (as discussed in the Discussion section).

**Fig 7:**
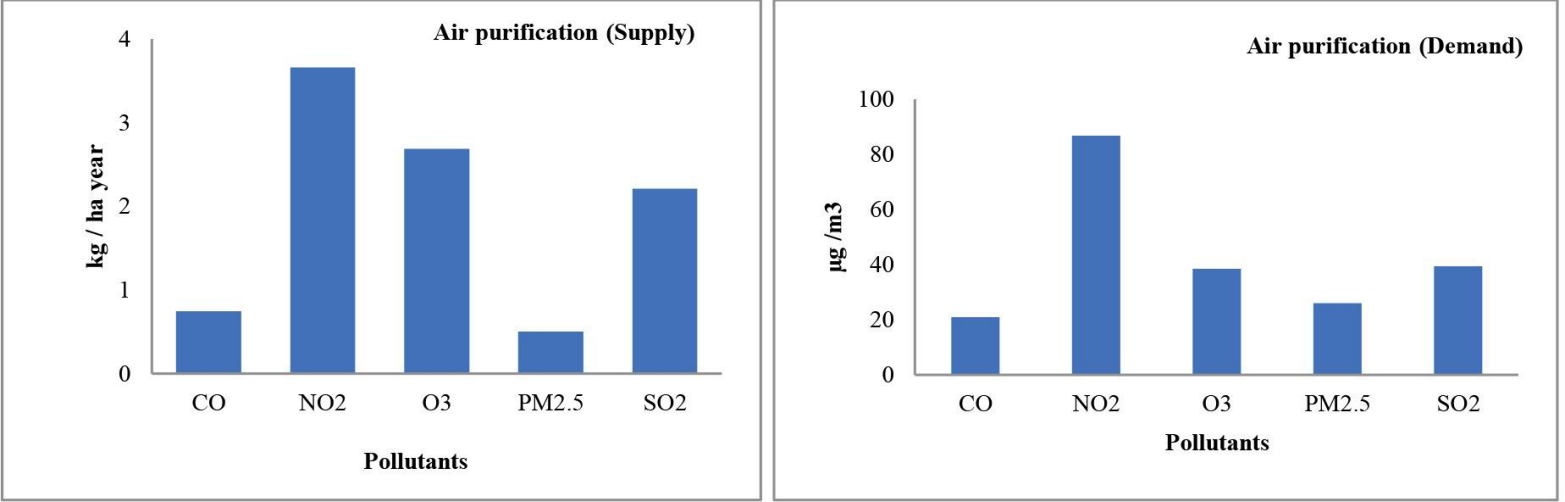
Results for air purification supply and demand (the value of CO in supply diagram is based on thousands) indicators for the case study.

The highest demand for URES air purification was associated with NO_2_, followed by SO_2_ and O_3,_ whereas the related supply indicators were highly similar between the pollutants. PM2.5 had the lowest removal rates among all the pollutants (Fig 7).

### Supply and demand for global climate regulation

Trees and shrubs in Tabriz sequester 29.20 thousand tons of carbon, which corresponds to about 1.4 t ha^−1^ year^−1^. In comparison, there was a demand for 674.32 tons of CO_2_-eq per hectare in 2015.

### Matches and mismatches

According to the results of the i-Tree Eco model, the expected annual mean levels of air pollution without air purification by urban trees or shrubs (as a hypothetic scenario) would not differ substantially from the current levels (Table 3). Therefore, the URES mismatch should be minor if realistic increases in URES supply are intended to meet the EQS. The results suggest that this situation occurs only for moderate mismatches. Also, the hourly air quality improvement for all pollutants by urban trees and shrubs over the year 2015 is shown in the appendix (S1 Appendix).

**Table 3:** Estimated air quality improvement due to air pollution removal by urban trees and shrubs.

	Pollutants				
	CO	NO_2_	O_3_	PM_2.5_	SO_2_
Annual mean air pollution levels with air purification delivered by urban trees and shrubs (μg m^−3^)	2089	86.79	38.39	25.93	39.34
Expected annual mean levels of air pollution with no air purification by urban trees or shrubs (μg m^−3^)	2090.03	91.775	41.523	25.703	42.476
Average percentage of air quality improvement (%)	0.0005	0.0575	0.0817	-0.029	0.0798
Amount of improvement (μg m^−3^)	1.02955	4.985	3.1375	-0.754	3.136

Hence, the contribution of urban trees to climate change mitigation is very low and accounts for about 0.2% of the overall GHG emissions, corresponding to a significant mismatch (Table 4). Following the framework in Fig 5, the matches and mismatches between the air purification service supplies and demands were identified (Table 4). Only in eight cases of air purification, the ES demand was not met by supply (Table 4). Here, we illustrate the procedure by discussing the results of the assessment of NO_2_ regulating services with respect to the WHO standard; The WHO annual limitation value for NO_2_ is 40 μg m^−3^ and the annual NO_2_ concentration as measured by air pollution monitoring station was 86.79 μg m^−3^. Hence, there was a 46.79 μg m^−3^ exceedance of the WHO value. The amount of air quality improvement for NO_2_ by urban trees and shrubs was 4.98 μg m−3 per year. Thus, the contribution of UF to reduce the WHO exceedance (10.62%) is lower than 20%, and a significant mismatch is identified.

**Table 4:** Results of assessing the matches and mismatches between URES supply and demand. “M” stands for matches, “S” for significant mismatches and “-” indicate that the assessment was not done because there was no EQS limit value.

URES	EQS	Pollutant					
		CO (0.0005*)	NO_2_ (0.058*)	O_3_ (0.082*)		PM_2.5_ (-0.03*)	SO_2_ (0.08*)
Air purification	WHO	-	S	M		S	M
	EU	M	S	M		S	S
	EPA	M	M	M		S	M
	IRAN	M	M	S		S	M
Global climate regulation	GHG reduction target	Contribution to GHG reduction targets					
		Unconditional			Conditional		
		S			S		

*: Average percentage of air quality improvement (%) by UF. These values are shown here to explain the relation between the air quality improvement provided by UF and the matches and mismatches. In other words, it helps to translate the magnitude of air quality improvement of each pollutant to level of mismatches or matches according to the associated EQS. For example, the 0.0005% improvement of CO resulted in matches according to all EQS.

The results of the match and mismatch assessment of the URES air quality improvement showed a huge variation among the different pollutants. None of the assessment standards of EQS values were met for PM_2.5_, which is in fact due to the negative improvements; the air quality improvement value for PM_2.5_ was -0.03% (as discussed below) (Fig 6). Hence, PM_2.5_ can be considered the most problematic pollutant in Tabriz. In contrast, CO seems the least problematic pollutant in the study area. Tabriz did not comply with Iran’s standard for O_3_ and the EU reference value for SO_2_. NO_2_ levels were below the EPA and Iran’s regulations but above the EU and WHO limits.

## Discussion

One of the essential issues required to further develop and implement the ES concept is to understand and assess the matches or mismatches between ES demand and supply in urban settings [13,102,103]. An important asset of our study was that we not only considered the supply side (i.e. the air quality improvement and climate change mitigation provided by urban forests), but also explicitly accounted for the actual demand of human societies for these regulating services provided by UF. Without such understanding of both aspects, misleading conclusions may be drawn about the benefits of these services [34]. Such understanding is also needed for urban planners to ensure optimum use of the current ES supply to meet the human demands throughout the city [34] and to make better decisions, i.e., a better match, in the future by appropriate urban design.

In this study, we assessed the matches and mismatches for two urban regulating ecosystem services: air quality improvement and global climate change mitigation. The present assessment for Tabriz, Iran exemplifies and concretizes the differences in demands and supplies between the two URES. Since the relationship between URES supply and urban deweller’ benefit is very complicated [9], we adopted a methodology based on indicators that provide a relatively simple, but still realistic, estimation of URES demands. As for indicators, we adopted a novel methodological approach using EQS as proxy indicators for the demand side. Our approach contrasts with other more time consuming and resource intensive assessment approaches such as socio-cultural elicitation [104]. This quick assessment approach can help policymaker to find out the condition of matches or mismatches between URES services supply and demand, and to analyze the potential of UF to overcome the mismatches.

Our results on air purification supply indicate that the mean air quality improvements were relatively small. The four different EQS gave slightly different results for some pollutants. For instance, matches were identified for NO_2_ when using EPA and Iran standards, while mismatches occurred when applying EU and WHO standards. These differences in assessing the regulation of the same ecological pressure may cause uncertainties and demand a better reflection on which air quality standards may be most reliable and adequate for a given situation to point out the actual human demand for clean air. In this case, it is expected that the WHO standards probably express a more reliable level of demand for air quality [105], even though only the Iranian air quality standard is legally binding within the case study. Particularly for NO_2_ and PM_2.5,_ there were major mismatches in supply and demand. The results even suggested some negative air quality improvement (%) values for PM_2.5,_ which means trees resuspended more particles than they removed (see S2 Appendix). Such negative values for PM_2.5_ occasionally occur in arid climates or areas experiencing drought. The PM_2.5_ deposition process is mostly mechanical involving the particulates being deposited on leaf surface during times of low wind. The reason is that the PM_2.5_ is not "locked-up" by the tree and can be redistributed into the air from the leaf surface areas if the wind is strong and there is no rain. In those cases, trees are actually facilitating the redistribution of particulates causing a negative impact of trees on PM_2.5_ [106]. Since the pollution removal value is based on the change in pollution concentration, it is possible to have situations when trees remove PM_2.5_ but increase concentrations and thus have negative values during periods of positive overall removal (for more information see [91]). In our study, such conditions are likely to occur at the combination of very low precipitation, drought and strong winds. However, even at conditions with a positive impact–i.e. when pollution levels are high–the mean contribution of URES supply to comply with the air quality standards is modest. Hence, there is a strong mismatch in supply and demand for air quality improvement.

Also, the contribution of the urban trees to climate change mitigation (supply) shows a very modest contribution compared to GHG emission (i.e. the demand for climate regulation services by urban forests). The reasons for this low contribution, or large mismatch, can be the relatively high emissions, urban morphology, the low level of urban forest (tree cover of 9.4%) and the young age of the trees (77.9% of the current trees are not grown enough to provide substantial URES [107,108]). There are various uncertainties in quantifying the studied URES supplies and demands: i) indirect effects of urban forests on climate mitigation, ii) GHG emission estimations were based on country-based estimations that included several emission sectors that were not within the study area, and iii) the applied i-Tree Eco model also has limitations in estimating the air pollution removal and carbon sequestration[see 36,68,109,110].

However, given the major mismatch, these uncertainties are unlikely to change the results significantly. Hence, as with the previous URES, UF have a limited potential for providing direct carbon sequestration to obtain the GHG reduction targets and to substantially mitigate global climate change (in line with [14,111].

These results clearly show that it is insufficient to focus only on the supply of ecosystem services, i.e. to promote urban greenery based on the argument that it provides important services. In addition, because both supply and demand are likely to be temporally dynamic features, the development of the matches and mismatches needs to be monitored continuously. For instance, as the trees grow, the supply of regulating ecosystem services is likely to increase substantially. In this context, it will also be important to evaluate whether the actual amount of ES supply remains sustainable and can be provided over a long period of time [71]. This aspect is not often taken into account [112], and it is recommended to develop indicators that additionally show whether the provisioning of ecosystem services is sustainable.

Although there is currently no consensus on which comprehensive approach to use for integrating the ES concept in the urban planning process to maintain proper urban sustainability, efforts such as assessing mismatches between the actual demands of human societies based on the supply of urban ES may constitute an important component of such approaches. Therefore, our analysis provides important insights for urban environmental planners, in Tabriz and other cities. Analyzing the potential of UF to mitigate carbon in comparison with the urban GHG emissions helps cities to evaluate whether the GHG reduction goals are met or not [111,113]. Such an analysis is important, because there are often limited urban resources to implement urban environmental strategies such as GHG reductions [114,115], and quantitative analyses such as the one provided by this study can provide a leverage to get more urban resources allocated to (regulating) the ecosystem services. Currently, there is a very limited amount of such quantitative scientific documentation to support the city planners in evaluating the effectiveness of ES based programs in cities [111,115]. For instance, the Department of Environment of Iran, which is responsible for meeting the regulatory requirements (e.g. air pollution mitigation), currently faces many difficulties in implementing an ecosystem service approach; and our approach may fill this critical gap.

The main purpose of this approach is to make the mismatch results applicable for policy- and decision-makers. Therefore, we converted the quantitative results to modest and significant mismatch classes. Moreover, even if urban forests have only a modest and maybe a negligible, contribution to providing important regulating ecosystem services, they may provide a complementary measure in a total set of measures to mitigate air pollution and GHG emissions, as even such small effects can have significant health benefits [68,116,117] and also supply other environmental and economic benefits (e.g., noise pollution reduction, aesthetic benefits, etc.) [118,119]. It means determining which categories of mismatches can be solved by UF developing. In other words, the modest mismatches suggest that they can be overcome partially by urban greenery which is intended to increase the ES supply, but the significant mismatches suggest that EQS can be unlikely met by the increment in ES provision through green space development programs, and needs more extensive action than only urban greenery strategies.

For Tabriz, the major mismatch in the regulating services shows that Tabriz struggles to comply with the current regulating ES demands. This mismatch suggests that what is needed to decrease the mismatch is probably both a major investment to reduce the rate of pollution and carbon emissions and a major increase in the provision of URES through urban greenery. The latter would be more cost-effective if synergies are considered along with other urban ES. This will demand a more holistic and comprehensive analysis than the one applied in this study.

Assessment of URES supplies and demands requires matching the data with the same spatial scales [34]; however there is often no such consistency [120]. Generally, URES supplies and demands are spatially explicit [13,121,122]. Also, clear localization of URES demands is usually impossible and occasionally unreasonable, which is principally due to the lack of a final good or end-product [25]. It means that temporal, spatial and stakeholder dimensions have to be taken into account to sufficiently inform urban sustainable decisions [1]. Also, in determining the supply and demand realization, it is of particular importance to spatially distinguish and consider the URES flow processes and pathways which carry services through the landscape from the (excess) service providing area to the (excess) service-benefiting area. These two areas can be identical, overlapping or separated [34,101,123].

In solution-oriented studies, focusing on site-specific solutions is acknowledged [124], and thus precise and reliable data are required [102]. Therefore, as the scale of our analyzes match the scale of urban decision making (municipal scale), the results can be helpful in assisting urban planners and policymakers to proactively concentrate on specific and more sustainable solutions and scenarios [124].

## Conclusions

Our approach, which is based on indicators of ecosystem services, allows assessing the matches and mismatches between URES supply and demand. Compared to the existing approaches, the application of proxy indicators is relatively straightforward. The results of this assessment can be used for future urban strategies and planning as well as for scenario analyses informing such planning. Such analyses will show whether the demands of urban dwellers for URES can be met sustainability through the prevailing ES capacity of UF or if other measures (e.g. technological alternatives or substantially improved urban greenery) are required.

We suggest that the applied framework may provide a simple, though flexible, approach that has the potential to improve the applications of ES concept in science as well as in practice in the study area and other cities all over the world for urban sustainability management.

## Supporting information

S1 Appendix Hourly air quality improvement by urban trees and shrubs regards air pollutants. (PDF)

S2 Appendix PM2.5 flux (Dry deposition) per unit tree and shrub and covers. (PDF)
