# Supplementary material for: An improved method for assessing mismatches between supply and demand in urban regulating ecosystem services: A case study in Tabriz, Iran
Source: PLoS One. 2019 Aug 15;14(8):e0220750. doi: 10.1371/journal.pone.0220750 (PMC6695181; doi:10.1371/journal.pone.0220750)
Supplement: S1 Appendix — (PDF) [file pone.0220750.s001.pdf]

S1 Appendix: Hourly air quality improvement by urban trees and shrubs regards air pollutants

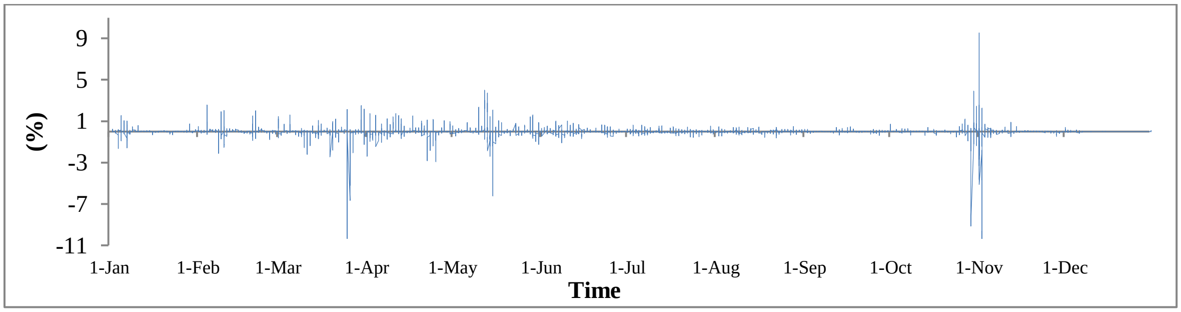

Fig A. Hourly air quality improvement by urban trees for PM2.5 (%) during 2015

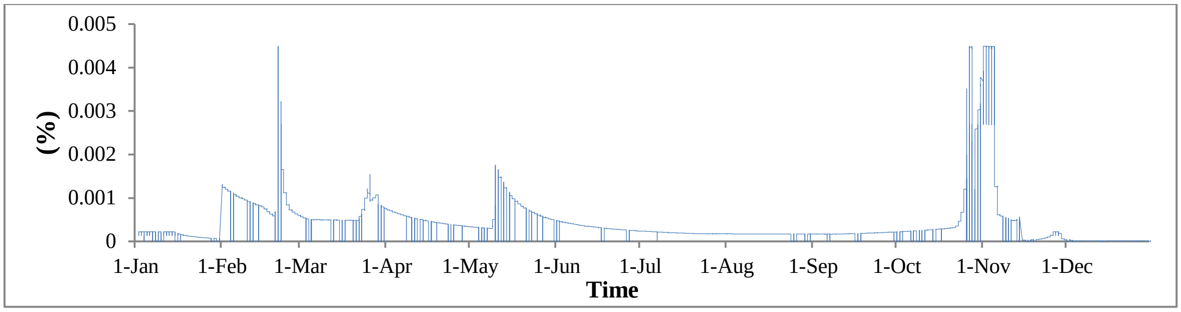

Fig B. Hourly air quality improvement by urban trees for CO (%) during 2015

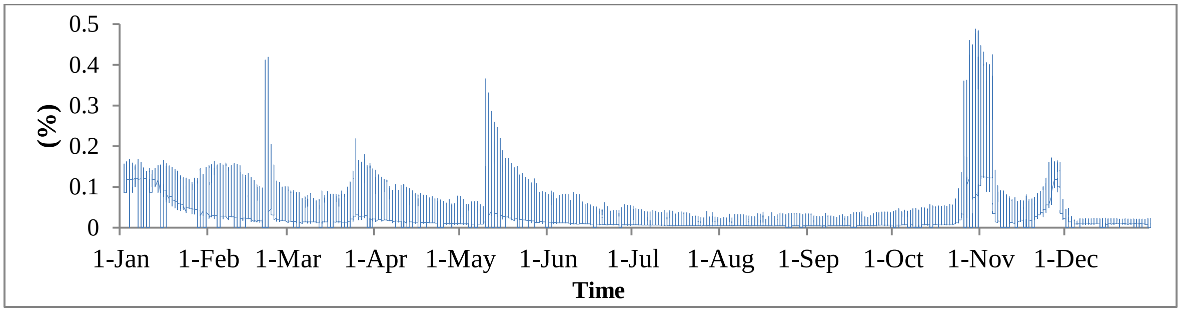

Hourly air quality improvement by urban trees for NO2 (%) during 2015

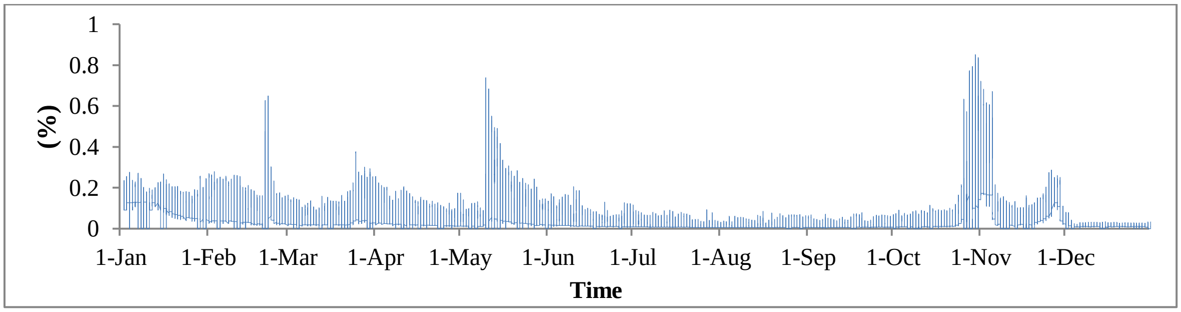

Fig C. Hourly air quality improvement by urban trees for O3 (%) during 2015

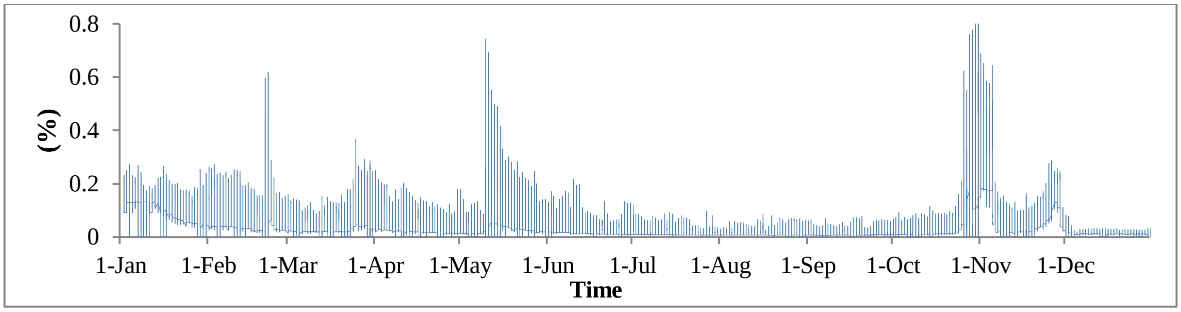

Fig D. Hourly air quality improvement by urban trees for SO2 (%) during 2015

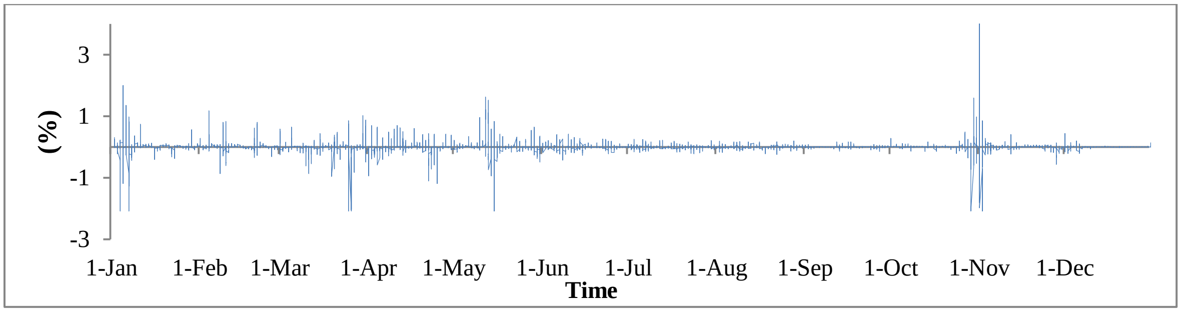

Fig E. Hourly air quality improvement by urban shrubs for PM2.5 (%) during 2015

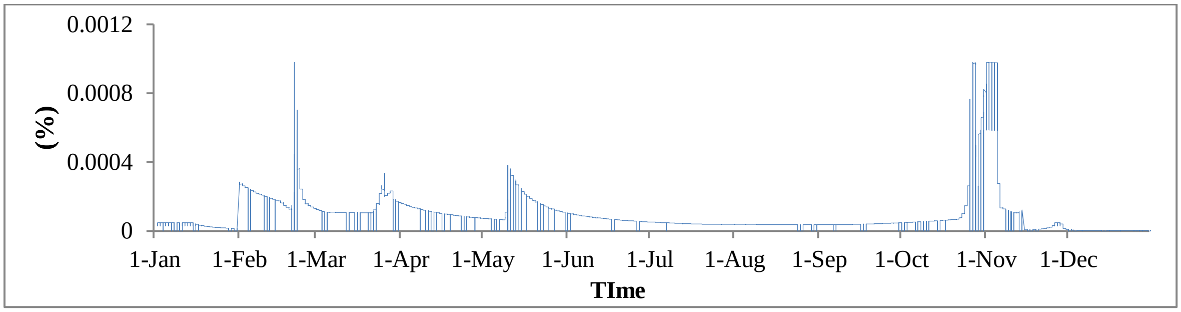

Fig F. Hourly air quality improvement by urban shrubs for CO (%) during 2015

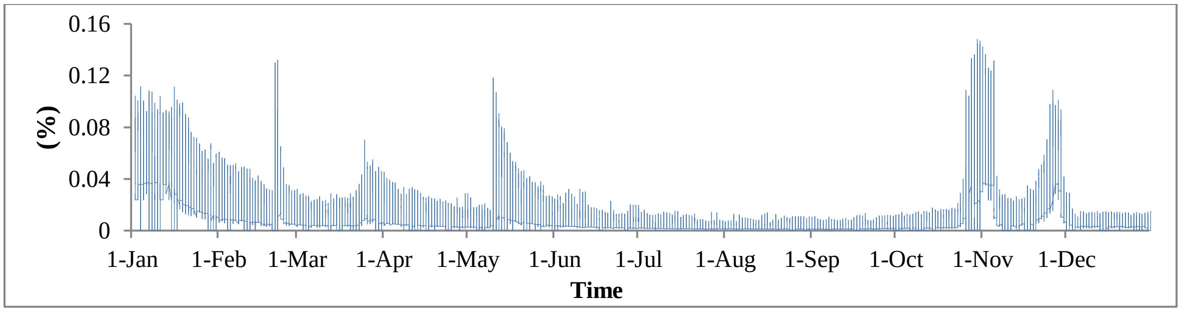

Fig G. Hourly air quality improvement by urban shrubs for NO2 (%) during 2015

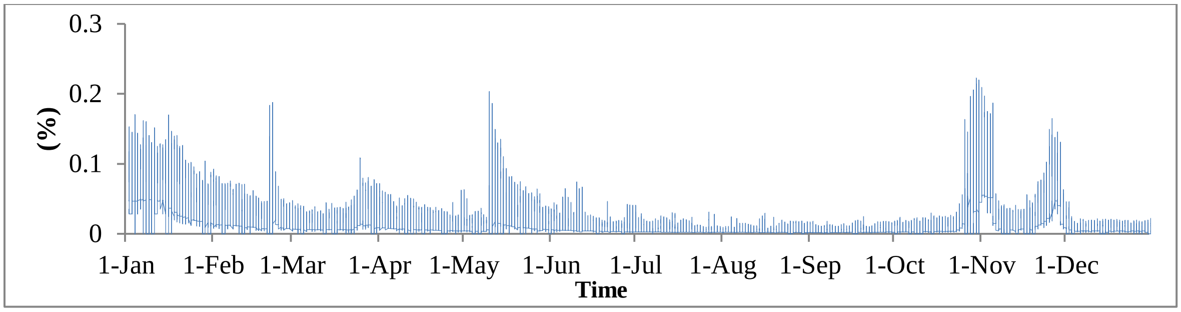

Fig H. Hourly air quality improvement by urban shrubs for O3 (%) during 2015

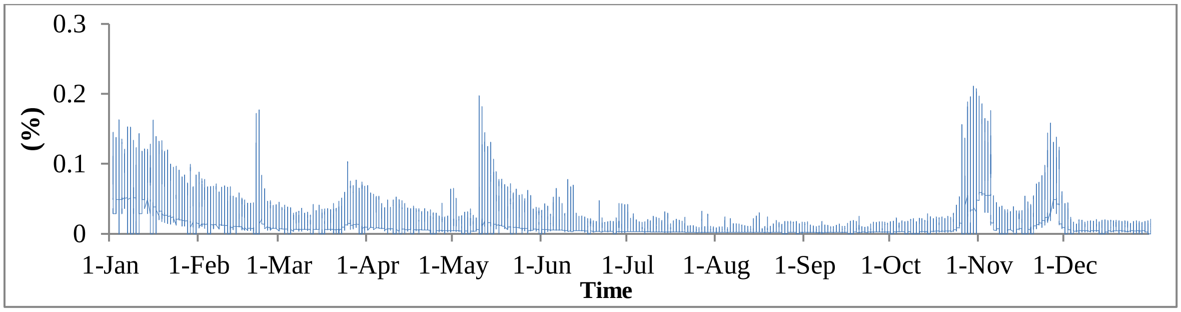

Fig I. Hourly air quality improvement by urban shrubs for SO2 (%) during 2015
