# Supplementary material for: An improved method for assessing mismatches between supply and demand in urban regulating ecosystem services: A case study in Tabriz, Iran
Source: PLoS One. 2019 Aug 15;14(8):e0220750. doi: 10.1371/journal.pone.0220750 (PMC6695181; doi:10.1371/journal.pone.0220750)
Supplement: S2 Appendix — (PDF) [file pone.0220750.s002.pdf]

S2 Appendix: $PM_{2.5}$ flux (Dry deposition) per unit tree and shrub and covers.

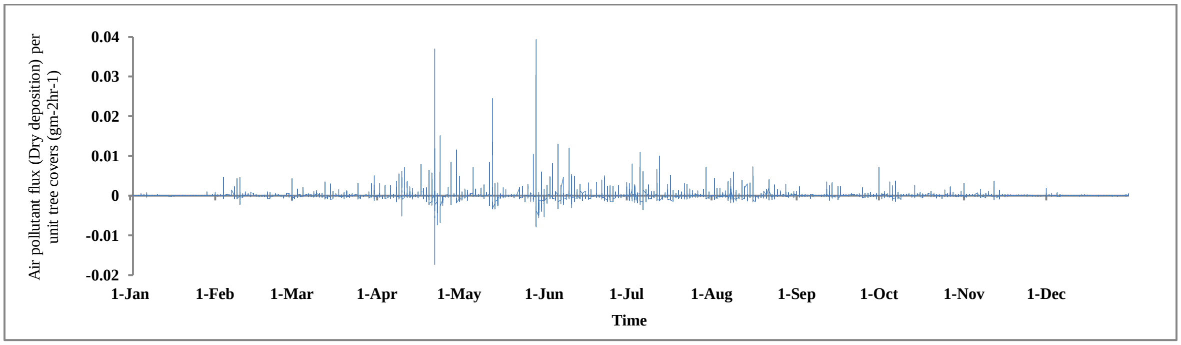

Fig A. $PM_{2.5}$ flux (Dry deposition) per unit tree covers ($gm^{-2}hr^{-1}$) during 2015

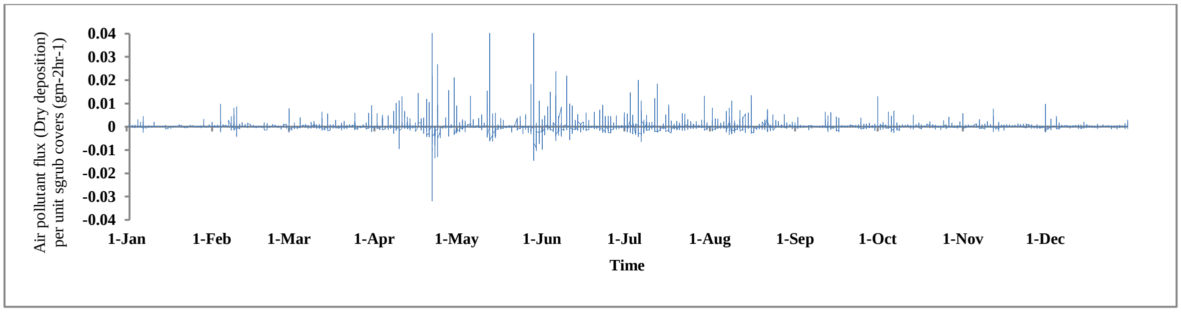

Fig B. $PM_{2.5}$ flux (Dry deposition) per unit shrub covers ($gm^{-2}hr^{-1}$) during 2015
